# Impacts of the COVID-19 Pandemic on Mobile Produce Market Operations: Adaptations, Barriers, and Future Directions for Increasing Food Access

**DOI:** 10.3390/ijerph191811390

**Published:** 2022-09-10

**Authors:** Anne E. Lally, Alban Morina, Leah N. Vermont, Jill N. Tirabassi, Lucia A. Leone

**Affiliations:** 1 Department of Community Health and Health Behavior, School of Public Health and Health Professions, University at Buffalo, Buffalo, NY 14214, USA; 2 Department of Anthropology, The College of Arts and Sciences, University at Buffalo, Buffalo, NY 14261, USA; 3 Department of Family Medicine, Jacobs School of Medicine & Biomedical Sciences, University at Buffalo, Buffalo, NY 14203, USA

**Keywords:** mobile produce market, food access, COVID-19 response, food retail

## Abstract

Background: Mobile produce markets were increasingly recognized as an effective and accepted approach to improving access to fruits and vegetables in lower-income and at-risk communities during the first year of the COVID-19 pandemic in the United States. This study provides insights into how mobile market operations were impacted by, and evolved in response to, challenges posed by the pandemic. Methods: A survey evaluating impacts of the pandemic on mobile markets was distributed to a database of mobile market operators in the United States. Respondents were asked to describe impacts to their mobile market’s operations, and what adaptations were needed to continue to effectively serve their communities during 2020. Results: Surveys representing 48 unique mobile markets were collected from March to July 2021. Of the respondents, 63% reported an increase in demand for mobile market services from community members. Furthermore, 65% increased the amount of produce they distributed in 2020 as compared to 2019, often through adopting low or no-cost models or participating in pandemic government programs. Discussion: Emergency adaptations employed by mobile markets can inform long-term operational modifications for not only mobile markets, but also other food access programs, beyond the COVID-19 pandemic.

## 1. Introduction

The coronavirus disease of 2019 (COVID-19) pandemic and its reverberatory effects on the economic and social landscape of the United States increased food insecurity among and across at-risk populations. National data collected in 2020 found that food insecurity doubled overall in the United States, with households with children reporting food insecurity at triple the rate of previous years [1]. The rate of American adults reporting that members of their households sometimes or often did not have enough to eat ballooned from nearly 8 million adults (4%) in 2018 to 26–29 million (11%) during April to July 2020, coinciding with an increase in economic vulnerability (unemployment or job insecurity) as a result of the pandemic [2]. Millions of additional individuals enrolled in food and nutrition assistance programs administered by the U.S. Department of Agriculture (USDA) in fiscal year 2020, with many of these programs evolving and expanding in response to the pandemic [3]. Groups already experiencing disparities in food access caused by historical inequities and structural oppression, including Black, Indigenous, and People of Color (BIPOC) individuals and families, have been disproportionately impacted by the interconnected effects of unemployment or economic vulnerability and food insecurity during the pandemic [1,2,3,4,5,6,7,8].

Pandemic-related safety measures implemented in 2020, including social distancing and the closure of businesses deemed non-essential, also had profound effects on food-related behaviors for individuals and families; most notably, a shift away from eating outside of the home (restaurants, institutional food service, etc.) and an increase in cooking and eating in the home [5,9,10,11,12,13,14,15,16,17]. International research on early pandemic eating habits indicated a decrease in fresh fruit and vegetable consumption, increased unhealthy food consumption, as well as decreased food diversity during pandemic lockdown restrictions [12,13]. However, subsequent studies indicated a shift towards more cooking and eating at home has created a context for an increase in healthy eating behaviors, including increased fruit and vegetable consumption, for individuals and families [9,10,11,14,15,16].

The ability to adopt healthier eating behaviors was largely governed by access to food retailers viewed as safe by consumers, as well as the ability to order groceries online for pick-up or delivery [5,14,17]. Restaurant and other food service (e.g., schools and hospitals) closures shifted where people purchased food and put a strain on retail food outlets and supply chains [5,18,19,20,21,22]. This presented major challenges for lower-income communities and those with limited access to full-service food retail stores, particularly those that offer healthy foods like fruits and vegetables. Although fast food consumption dropped slightly overall during the pandemic, higher levels of fastfood consumption were reported among socioeconomically disadvantaged adults [23]. Additionally, consumer trends of stockpiling goods led to shortages that impacted individuals unable or unwilling to participate in these behaviors, putting lower income individuals at greater risk of not being able to access food in an already strained food retail environment; stockpiling behavior was more often correlated with higher income households [16,17].

In response, many nonprofits and food relief organizations stepped up to fill the gap in healthy food access [4,18,24,25,26,27,28,29,30]. Mobile produce markets are one such solution that has been growing in popularity [31,32,33], especially within the context of the COVID-19 pandemic. Mobile produce markets, also referred to as mobile markets, are small fruit and vegetable markets that travel to communities with reduced access to traditional food retail [31,32,34]. Mobile markets work to increase affordability of healthy foods through nutrition incentive programs and strategic pricing, and typically provide customers with nutrition education [31]. Previous research indicates that mobile markets can increase fruit and vegetable consumption in under-resourced communities [32,33,35,36,37,38,39,40,41,42]. Given their high potential for improving diet, low start-up cost compared to brick-and-mortar retail, inherent mobility, and focus on meeting the food needs of underserved communities, mobile markets emerged as a common approach to address food access challenges during the early stages of the COVID-19 pandemic.

The goal of this research is to understand the role of mobile markets in local food systems that were thrown into flux by the COVID-19 pandemic, and how the unique operational models of mobile markets allowed for these programs to evolve swiftly in response to challenges faced by food retail and food access programs during the first year of the pandemic. We identify adaptations that mobile markets made in response to the COVID-19 pandemic using data collected from mobile markets across the United States (US). This research can be used to better understand the characteristics inherent to mobile markets, as well as adaptations specific to the COVID-19 pandemic, that make this food access intervention an important tool for increasing food access during public health and natural disasters.

## 2. Materials and Methods

### 2.1. Study Design

This study used a mixed-method approach to understand how mobile market operations were impacted by the COVID-19 pandemic. Using an exploratory sequential mixed methods design, content analysis of qualitative data derived from a series of open calls for mobile market operators held from April through November 2020 was used to design a web-based survey. The survey was completed by mobile market operators across the United States. Respondents were asked to complete the survey based on their organization’s mobile market operations. Qualitative data was also collected using open-ended survey questions, and content analysis was completed to summarize the results and provide further context for the quantitative responses. The purpose of this mixed-method design was both to inform the development of the quantitative method using qualitative data and also to provide a more complete representation of the quantitative responses [43]. Mobile market operations possess a level of variability that is best represented using a mixed-method approach to capture generalities as well as unique responses.

### 2.2. Participant Recruitment

Participants were recruited from the mobile market contact list of the Veggie Van Training Center (VVTC), which included 515 individuals at the time of survey distribution. The Veggie Van Training Center, based at the University at Buffalo, is the backbone organization for the Mobile Market Coalition and provides training and support for mobile market operators across North America. The contact list was developed through registration for the Mobile Market Summit, an annual conference started in 2019 to engage the mobile market community, as well as through registration to access the Veggie Van Toolkit, an evidence-based resource for planning and operating mobile markets. Individuals on the contact list include those operating mobile markets, those interested in starting a mobile market at their organization, those working in academic and professional fields relevant to mobile market operations, and those engaged in similar food access and health equity community-based work. Participants were also recruited from the 231 mobile market operators who are part of the Mobile Market Network listserv. A series of emails soliciting participation were sent to the 515 individuals on the mobile market contact list and the 231 operators included on the Mobile Market Network Google Group in late March through early April 2021. The survey was also included in the 2021 Annual Mobile Market Survey disseminated by the VVTC in June through July 2021 to capture additional participants. Participating individuals completed the survey from 26 March to 7 April 2021 and from 14 June to 19 July 2021. Individuals had to be employed at an organization that operated a mobile market at the time of distribution to be eligible to complete the full survey. The survey responses from individuals who reported working at an organization in the planning or exploratory phases of mobile market implementation were terminated after the demographic questions were completed and were not included in the full analysis. The Annual Mobile Market Survey, including the COVID-19 survey, was deemed exempt from IRB review by the University at Buffalo Institutional Review Board.

### 2.3. Survey Development

The survey was developed using COVID-19 related measures from similar organizations, in addition to measures tailored specifically to MMs based on content analysis of qualitative data collected from mobile market operators during open calls held during the pandemic. We reviewed surveys with similar goals distributed to farmers markets, food retailers, and food access organizations. The Duke University World Food Policy Center’s “Impact of COVID-19 on Hunger Relief Organizations 2020 Survey” [44] and the Farmers Market Coalition’s “COVID-19 Impacts Summer 2020 Survey” [45] informed the design of the survey. Questions on revenue generated in the summer of 2020 were modeled after the Farmers Market Coalition “COVID-19 Impacts Summer 2020 Survey.” Subsequent survey items describing reasons for changes in revenue were adapted to the operational setting of mobile markets based on qualitative data previously collected from mobile market operators. Questions on operational barriers from the Duke University World Food Policy Center’s “Impact of COVID-19 on Hunger Relief Organizations 2020 Survey” were also adapted for mobile market operations.

Qualitative data collected from the Mobile Market Network Open Calls on COVID-19 informed how questions were tailored to the mobile market setting, most importantly the variables provided for operational questions that were based on other, more general, food access questionnaires. In April 2020, the VVTC coordinated with members of the Mobile Market Network to host weekly Zoom meetings for mobile market operators to share resources, discuss barriers, and crowd-source solutions to novel issues resultant of the COVID-19 pandemic. Hour-long sessions were scheduled weekly from 15 April to 23 November 2020 and provided both an open forum for discussion as well as presentations from mobile market operators. Call attendance varied from 4–38 operators. Topics of discussion included: designing revised operational plans in adherence to local and state pandemic restrictions, new funding opportunities for food access programming, home delivery models, pre-ordering and associated technology, virtual community outreach, sourcing during the earliest stages of the pandemic, free and donation-based models, managing volunteers and staff remotely, enforcing mask mandates, and preparing for the Winter 2020 market season while observing social distancing. Sessions were recorded and summarized as memos by an undergraduate research assistant. We used the feedback from these open calls to identify predominant topics to address quantitatively amongst mobile market operators through the “Impact of COVID-19 on Mobile Markets in 2020 Survey.” Draft survey questions were reviewed by two community-based mobile market operators for clarity and relevance prior to final revisions and dissemination.

### 2.4. Survey Questions

The “Impact of COVID-19 on Mobile Markets in 2020 Survey” (see Supplementary File) focused on the following five key areas related to mobile market operations during the first year of the COVID-19 pandemic: (1.) mobile market organization demographics, including organization name, location, operational status, how long the market had been operating, and urbanicity of locations served; (2.) communities served during the 2020 season, including community demand, season length, and number of sites visited; (3.) impacts of COVID-19 on market organization revenue, including annual revenue compared to the prior year, reasons for reduced revenue, efforts to offset lost revenue, and overall produce distributed; (4.) impacts of COVID-19 on fruit and vegetable incentive program usage at mobile markets, including participation in, and uptake of, pandemic relief programs and nutrition incentive programs. Emphasis was placed on the USDA Farmers to Families Food Box (F2F) program, which funded the purchase of agricultural products to be packaged into family-sized food boxes that were distributed by community organizations [46], as mobile market operators applied for and received contracts to execute the program in varying capacities; and (5.) operational changes made in response to COVID-19 restrictions, including questions about what types of operational pivots or accommodations markets made and if these changes would be adopted long-term.

### 2.5. Data Analysis

Statistical analysis was conducted using IBM SPSS Statistics for Windows, version #28.0 (Armonk, NY, USA: IBM Corp). Prior to analysis, we removed duplicate responses (e.g., more than one person responding from the same organization), keeping the organization’s most complete set of responses for analysis. For those organizations with multiple complete responses, we included the response that was recorded first. Organizations that were not operating a mobile market at the time of survey distribution were terminated after completing demographic questions and were not included in analysis. Frequencies and percentages were calculated for categorical variables. Free-form text responses recorded as “other” were reviewed and counted as categorical variables when applicable; “other” responses that could not be categorized as variables were grouped into common topics and reported as frequencies where relevant. Data was also analyzed to check for differences based on length of mobile market operation (<2 years vs. 2+ years). Pearson chi-square tests were completed using a designated alpha level of 0.05 to test for significance of associations. We completed descriptive statistics to summarize responses for the following areas: community organization demand for mobile market services, customer demand for mobile market services, effects of the pandemic on operations and funding, effect of the pandemic on distribution, and increase or decrease in fruit and vegetable incentive program uptake between the 2019 and 2020 seasons.

The responses to two open-ended questions on pandemic-related barriers and operational pivots employed in response to the pandemic, but adopted long-term, were grouped into main themes using conventional content analysis. Frequencies, where applicable, were reported descriptively.

## 3. Results

### 3.1. Mobile Market Organization Demographics

We received 118 total responses to the “Impact of COVID-19 on Mobile Markets in 2020 Survey.” While only one response was encouraged from each organization, some participants employed at the same organization started one or more surveys, resulting in 34 duplicate responses that were removed from analysis; in these instances, the most complete survey and/or the survey completed first sequentially was recorded. We excluded 27 markets that were not currently operating, including those that were actively working on setting up a mobile market (n = 11), that had previously explored options for starting a mobile market (n = 2), that were interested in learning more about mobile markets (n = 7), that had operated a mobile market in the past but not currently (n = 1), or that did not provide a response to this question (n = 6). Incomplete surveys (n = 9) and those with no market description provided (n = 6), were also removed. After removing duplicates and ineligible responses the data represented 48 mobile markets. Eligible organizations represented 23 states, with 35% from the Northeast (n = 8), 26% from the Midwest, 17% from the West, and 22% from the South. Of these markets, 19% had been operating a mobile market for less than two years, and 81% had been operating a mobile market for more than two years. In terms of populations served, 88% served urban populations, with 44% serving rural populations, and 35% serving suburban populations(see Table 1).

### 3.2. Communities Served during 2020 Season

Most surveyed mobile markets experienced an increase in customer demand for their services in 2020. A total of 63% reported seeing more demand for a mobile market program amongst the customers they served, with 25% reporting about the same demand for a mobile market program. In addition to individual customers, respondents were surveyed on perceived demand for their mobile market from community organizations requesting to host new or additional market stops. The majority of survey respondents (58%) reported an increase in demand for mobile market services from community organizations and established community partners, while 29% reported no change in demand from community partners in 2020 as compared to previous years (see Table 2).

A total of 48% of the surveyed organizations reported operating a mobile market for four to seven months in 2020. Markets with a year-round season represented 21% of organizations surveyed, followed by 19% that operated a market for eight to 11 months, and 12% that reported a season less than four months long. Although 35% of surveyed markets reported maintaining the same season length during the first year of the pandemic, 25% reported a decreased season length in 2020 compared to prior years. Despite an increased demand for mobile market services, only 19% reported being able to increase the length of their mobile market season (see Table 3).

Of surveyed markets, 40% visited fewer market sites during 2020, 29% visited more market sites, and 12% visited approximately the same number of sites as the previous year (see Table 4). Some markets were able to serve the same number of sites by changing their operational models at some or all of their sites to accommodate restrictions and related time, space, and staffing constraints; these markets often shifted from a full-service market to pre-order and pick-up only models, as described by a respondent, “to safely serve [customers] when there was not enough room to abide by social distancing restrictions.”

For those markets that responded to the multiple-choice question on how they changed the number of sites they visited in 2020 (n = 31), prioritization of sites was based predominantly on community demand and access; 74% of surveyed markets identified community demand as a contributing factor in prioritization of operational sites. This included prioritizing existing partnerships and reducing new or pilot sites, as well as making the most effort to return to the “most affected sites.” Of the prioritized sites, 71% remained accessible in spite of pandemic restrictions (e.g., public parks, community organizations vs. schools, churches, municipal offices that moved to remote operations). Food insecurity data was used by 42% of the surveyed markets to decide which sites they should prioritize, while 13% informed their decisions using income data for communities served (see Table 5).

### 3.3. Impacts of COVID-19 on Market Organization Revenue

Of the markets surveyed, 37% reported a decrease in market revenue when comparing June, July, and August 2020 to the same period in 2019, and 17% of the markets reported an increase in revenue, with 15% reporting no change in revenue (see Table 6).

Of the 22 markets completing this question, the majority (77%) said that a decrease in customer attendance was the predominant reason for reduced market revenue in June, July, and August 2020, as compared to 2019. Only 9% of markets responding to this question experienced either a suspension in funding from their current funder(s) or a decrease in merchandise sales without a decrease in customers. Free-text responses recorded as “other” included 14% of respondents that pivoted to, or maintained, a free model; these organizations pivoted to a “free ‘mobile food pantry’” model, “completely shifted [their] model during these months… and applied for grants to be able to give away produce for free to several low-income communities,” or continued to follow a free distribution model (see Table 7).

Of those that implemented new financial approaches during the early pandemic period (65%), the majority infused their budgets with funds from existing, new, or COVID-19-specific grants or financial programs; see Table 8 for full results. Those respondents reporting “other” actions to offset lost revenue were able to balance reduced revenue by reorganizing staff and reducing costs. One respondent described how a “decrease in sales was almost balanced out by a decrease in staff time and inventory expenses. We definitely generated less revenue, but we spent less money, too.” Another respondent described how “staff were switched to other roles in the organization during the summer so new seasonal… market workers did not have to be hired in 2020.”

Of those respondents that had 2019 data to compare year over year distribution (n = 40), 65% reported an increase in produce distributed, 20% reported approximately the same produce volume distributed in 2020, and 15% reported a decrease in produce volume distributed in that time period (see Table 9).

Although the majority of respondents did not participate in the Farmers to Families Food Box Program, the 33% that were awarded contracts were able to do so based largely on the mobility and community partnerships inherent to mobile market operations; see Table 10 for full results.

### 3.4. Impacts of COVID-19 on Fruit and Vegetable Incentive Program Usage at Mobile Markets

Of all surveyed mobile markets, 58% reported an increase in fruit and vegetable incentive programs in 2020 and 25% reported no change or that they did not offer any of the incentives in 2020. See Table 11 for full results by incentive program.

### 3.5. Operational Changes Made in Response to COVID-19 Restrictions

Market operators were asked if their mobile markets allowed pre-orders or pre-payments by phone, text, or online, either as a response to the COVID-19 pandemic or as an existing policy. The majority (61%) did not accept pre-orders or sales online, by text, or by phone at the time the survey was completed. A total of 33% began offering pre-orders and or pre-payments as a response to the pandemic, and only 6% had done so prior to the pandemic (see Table 12); only markets operating for longer than two years offered pre-orders prior to the pandemic. Of those accepting pre-orders or sales online, by text, or by phone (n = 19), the majority (37%) accepted only online orders (n = 7); 21% accepted only phone orders (n = 4); 15% accepted both online and phone orders (n = 3); 11% accepted online and text orders (n = 2); 11% accepted online, text, and phone orders (n = 2); and 5% accepted text and phone orders (n = 1).

Respondents were asked to provide an open-ended response detailing barriers or challenges experienced by their organization’s mobile market because of the COVID-19 pandemic. Of the 33 responses collected, 33% described barriers related to accessing and/or changing their market locations due to social distancing protocols and host site shutdowns (n = 11) One respondent explained that “it was a huge challenge to figure out new schedules and protocols at each of [our market] locations based on what they were doing” since they partnered with community organizations to host their mobile market.

Another common theme identified by 30% of respondents was balancing safety protocols with customer service, and how finding this balance impacted their operations (n = 10). One respondent described how their market had difficulty “balancing the safety needs with customer needs… having to have more staff at the market and reduce the number of sites that we can attend.” Similarly, another respondent described how their market had issues with the “general safety of customers—[a] different shopping process caused us to restructure our staffing needs.”

Staffing challenges overall was another common theme for mobile market operators during the pandemic, with 24% describing challenges related to staffing and or volunteers (n = 8). One respondent described the individual impacts on staff, “safety precautions were extensive and draining on staff, [we] needed more staff but didn’t have funding for it.” Market operators also reported customer-level challenges, including reduced customers, and customer-level financial concerns. One respondent described how their “market is very people-centric, and not being able to closely interact with them was hugely limiting. Having less markets meant less exposure and less customer transactions and interactions.” Another respondent described how “reduced foot traffic at markets because of changes to local benefits programs” impacted their revenue. Respondents were also asked to provide an open-ended response about any innovations, operational “pivots” or adaptations that were implemented in response to the COVID-19 pandemic that they had adopted as long-term or permanent features of their market. The most common type of adaptation reported by the 37 respondents who provided responses was related to day-to-day market operations (46%, n = 17). Responses varied in their specificity due to the individual nature of market operations, but three respondents focused on staffing changes; one market operation would permanently adopt having “two staff working each market. [This is] easier on the body and safer,” another market would have a “dedicated register person” going forward, and the third described “lik[ing] having additional staff capacity at sites.”

Open-ended responses about adaptations that were adopted permanently fell into two main thematic areas in addition to the more general day-to-day operational changes described above: online ordering and or pre-ordering (22%, n = 8), and a produce bundle or box model (e.g., offering multiple produce items sold together at a reduced cost) in addition to, or instead of, a free choice market (22%, n = 8). Online ordering and or pre-ordering was often described as an imperfect solution; one respondent described how pre-ordering was “time consuming and not ideal, [but] the customers who do pre-order really like it. I believe it increased our revenue to have regular customers pre-order every week.” Similarly, another respondent stated, “we built out an online store for pre-orders that allowed us to bridge the gap at some business stops we couldn’t go to in person—it was mildly successful, but unclear if we will continue with it or not.” Four markets reported offering a produce bundle or box to facilitate a delivery program or encourage reduced time spent by customers at the market site. Three markets that adopted a produce bundle or box model reported doing so to offer greater value to their customers; one respondent described how their market “switched to creating a $5 bag, it is able to feed a family of four.” Other common COVID-19 adaptations adopted permanently by markets included: delivery models (n = 5), new forms of food sourcing and or production techniques (n = 4), enhanced safety protocols (n = 4), prioritizing market sites more thoughtfully (n = 3), and new approaches to nutrition education (n = 2).

## 4. Discussion

Vulnerable populations are more likely to suffer poorer health outcomes during pandemics and COVID-19 has been no exception [1,2,3,4,5,6,7,8,47], as the effects of the pandemic on the food environment exacerbated the existing food insecurity experienced in under-resourced communities. Therefore, guaranteeing food access and assistance to these populations has become a priority since the early stages of the pandemic in the US in 2020. Mobile markets emerged as a component of relief efforts on the local level through the implementation of federal, state, and local programs. Mobile market operations across the country flexed in response to pandemic restrictions to continue to serve individuals and families facing food insecurity as those numbers increased. Participation in the USDA Supplemental Nutrition Assistance Program increased by 14% in the second half of 2020 as compared to the first half, with an average of 42.5 million people participating per month [3].

In the earliest days of the pandemic, mobile markets were able to respond quickly to fill the gaps in the food system created by the pandemic, namely, food retail closures or restrictions, fear about safety in food retail settings, and ongoing barriers to accessing fresh fruits and vegetables in communities not served by traditional food retailers [16,17,19]. Early reports of mobile markets suggested they adapted well to the COVID-19 food climate, as getting fruits and vegetables into communities that needed them was always a priority [48]. Our research found that 63% of mobile markets experienced an increase in demand for their services from community members, and 58% experienced an increase in demand from community organizations; similarly, local reports from the spring of 2020 document how mobile markets noticed an increase in demand or need in the communities they served [49,50].

Due to their inherent flexibility, collaborative nature, diverse resources, and community connectedness, mobile markets evolved in tandem with local and state restrictions and CDC guidance on pandemic protocols. Mobile markets, like the Arcadia Center for Sustainable Food and Agriculture Mobile Market in Washington, D.C., were some of the first food retailers and/or food distribution programs to be approved to operate under modified operations [51]. The on-the-ground design of mobile markets translated readily to social distancing protocols; changes to day-to-day operations, like adopting a no-touch market and facilitating increased safety precautions (e.g., social distancing, hand sanitization, sanitization of high-touch surfaces), were reported by mobile markets operating during the earliest months of the pandemic in 2020 [48,49,50]. This aligned with our finding that 46% of markets changed their day-to-day operations during the pandemic and intended to maintain those changes. Changes reported from our survey and collected during the Mobile Market Network Open Calls on COVID-19 included moving market operations from inside a vehicle to outside, providing queues marked with six-foot distances, setting up an outdoor produce display with actual items picked from within the vehicle by staff, and or providing additional space at checkout to accommodate socially distanced transactions. Subsequent considerations included providing shaded areas for customers waiting in line, setting up handwash or hand sanitizing stations, and having staff or volunteers available to monitor social distancing protocols. Additionally, the collaborative nature of mobile market operations allowed for the quick dissemination of pandemic operational plans across markets via the Mobile Market Network Open Calls on COVID-19 and the Mobile Market Network listserv.

Mobile market season lengths vary across the nation due to a combination of factors, including growing seasons, weather, organizational missions (e.g., only selling locally produced fruits and vegetables in a region with a short growing season), market infrastructure and set-up, community demand, finances, and other support [31,34]. Similar to season length, number of sites visited by a mobile market during the season is indicative of its community impact and reach. Through our survey data and qualitative responses collected during the Mobile Market Network Open Calls on COVID-19, two competing factors were identified as contributing to the number of sites visited by mobile markets during the pandemic: An increase in community demand for mobile market services, and a decrease in feasibility caused by pandemic restrictions. We saw that most (60%) of the markets surveyed kept the same, or decreased, their season length and 40% decreased the number of sites visited, despite reporting increased demand for their market. As such, there existed a tenuous relationship between increasing market sites to meet increased demand and balancing operational constraints by reducing market sites since revised operational plans required more manpower and time to set up and close down at market sites.

A trend towards a decrease in revenue generated at mobile markets during 2020 (38% of markets reported a decrease in revenue compared to 2019) contrasts with findings from the Farmers Market Coalition’s 2020 survey of farmers market managers, as food product vendors at farmers markets reported an increase in fruit, vegetable, meat, dairy, and value-added sales [52]. However, the majority of mobile market operators reported an increased volume of produce distributed in 2020 (65%), in spite of reduced sales. Mobile market operators who participated in Mobile Market Network Open Calls on COVID-19 described the disparity between revenue generated and produce distributed at their markets as resultant of a shift from a paid market model to a sliding scale/donation-based model, or a completely free model. New funding sources and programs often contributed to a shift away from the original operational models of the surveyed markets, including integrating free produce distribution alongside, or in place of, the typical low-cost, incentive-supported sales model employed by mobile markets through local programs and food banks or the USDA Farmers to Families Food Box Program. The flexibility to quickly shift payment models in response to community need exemplifies the utility of mobile market models in addressing food access during emergency responses.

Similarly, mobile markets were able to employ strategies to offset some, or all, lost revenue and increased costs due to the COVID-19 pandemic without major impacts to their operations because of their unique status as a community service existing between traditional food retail and non-profit food access programs. Of the mobile market operators, 68% submitted new grant proposals to both existing programs and pandemic relief programming at state and local levels; similarly, 67% of Hunger Relief Organizations (HROs) that completed the Duke University World Food Policy Center’s Impact of COVID-19 on Hunger Relief Organizations 2020 Survey reported establishing relationships with new funders as a factor contributing to their success during the early months of the pandemic [53]. Regarding mobile markets, 35% increased funding from their existing grants, and overall HROs recorded an increase in funding during this time. Although mobile markets can fall under the umbrella of HROs, including food banks, food pantries, and anti-hunger advocacy organizations [53], they often exist between a food retailer and a true HRO, which could influence funding considerations when compared to a more familiar food access program like a food bank or food pantry. Having a for-profit component, where produce is sold at a reduced cost and incentive programs like SNAP are accepted as payment, can help support the sustainability of mobile markets [34]; however, access to funding for HROs during a disaster response like a pandemic can be stymied by this food distribution model.

Mobile markets typically serve lower-income communities at risk for food insecurity. To maintain affordability for their customers, mobile markets employ reduced-cost models, accept nutrition assistance benefits like SNAP/EBT, and/or participate in incentive programs (e.g., produce prescriptions, state SNAP matching programs, or market-based loyalty programs) [31]. The USDA Economic Research Service reported a historic high of $122.1 billion spent on domestic food and nutrition programs in FY 2020, an increase of 32% from the previous year. Furthermore, 39.9 million people participated in SNAP alone each month, with a 31% increase in program spending compared to 2019 due to increased enrollment and increased benefits through emergency SNAP allotments disbursed in the second half of 2020 [3]. Of the surveyed mobile market operators, 71% accepting nutrition incentive programs in 2020 reported an increase in SNAP/EBT sales, including P-EBT. At traditional farmers markets, only a 39% increase in SNAP/EBT sales was seen during the same period of time (June-August 2020) [52]; based on this data, it can be seen that mobile markets are able to reach lower-income individuals receiving SNAP/EBT benefits more effectively, especially during a public health emergency.

Mobile market operators reported plans to adopt specific pandemic response adaptations long-term, including allowing pre-ordering (22%) and implementing bundling or box programs (22%). Pre-ordering services became increasingly important during the pandemic due to social distancing mandates and business closures. Ensuring access to pre-ordering for delivery or pick-up to lower-income communities was necessary to promote equitable access to food during the pandemic as these services were largely available to, and adopted by, higher-income individuals [5,9,11,17,23]. Other HROs also reported plans to sustain online ordering for their programs going forward [53]. The implementation of bundling or box programs in a mobile market setting has been seen to improve access, affordability, and acceptance of fruits and vegetables, as well as contributing to an overall model of increasing fruit and vegetable consumption [35,36,37]; the use of food boxes as a pandemic response and/or alternative approach to increasing food access is also expanding [4,54]. Other HROs readily adopted the bundle or box model to adjust to reduced staff and increased need; 83% of food banks and 63% of frontline organizations shifted to prepacked boxes [53]. However, only 11% of food banks and 16% of frontline organizations intended to continue to offer pre-packed boxes [53].

The increased acceptability of food boxes by consumers and mobile market operators during the pandemic was influenced, in part, by the USDA Farmers to Families Food Box Program, announced on 17 April 2020, as a component of the Coronavirus Food Assistance Program. A total of 33% of surveyed markets participated in this program. The program mobilized the USDA Agricultural Marketing Service (AMS) to partner with distributors to purchase fresh produce, dairy, and meat products from domestic producers and package goods into family-sized boxes. In turn, these boxes were distributed to community organizations, food banks, and other non-profits serving food insecure families to distribute boxes in their local communities. The program ended 31 May 2021 and recorded the distribution of 173,699,775 boxes of fresh produce, milk, and cooked meats over the year (May 2020–May 2021) the program operated [46]. Capitalizing on the design of their mobile market vehicles to sustain heavy loads and refrigerate goods, 31% of those surveyed markets participating in the program delivered food boxes to other non-profit organizations in the community to disseminate, and 63% distributed food boxes outside of their mobile market sites, including home delivery, structured pick-ups, and or dissemination at community events. Of the surveyed markets participating in the Farmers to Families Food Box Program, 19% acted as aggregators; a contract that was difficult to secure due to requirements on storage, distribution networks, sourcing, and capacity. Only one mobile market operating for less than 2 years participated in the program as a distributor and zero markets operating for less than 2 years acted as aggregators, suggesting more established markets were better suited to participate in the program, especially as aggregators.

Limitations of the present study included a low response rate from mobile market operators when the survey was distributed in March to July 2021; this period of time is often busy for operators preparing for the busiest market season, and, for some, opening their market after closing for the winter season. Other limitations included the use of novel survey measures as standards for COVID-19 food access metrics, which have yet to be developed for food retailers and HROs. One strength of the study was contributions to the development of standardized survey questions within this space.

## 5. Conclusions

This research reveals the important role that mobile markets played in local food systems responding to the COVID-19 pandemic. This study offers a summary of challenges experienced by mobile market operators as they pivoted their operations to fulfill their common mission to provide healthy food access to communities facing food insecurity, especially as need increased and feasibility decreased. This study also provides data on adaptations made by mobile markets and their ability to support local and federal food access programs when traditional food retail operations were destabilized, deepening food insecurity in communities already at risk.

Future research should focus on the long-term sustainability of COVID-19 related operational pivots, how these changes impact revenue and program success in the coming years, and how mobile markets and other food access programs can be prepared to mobilize in future public health emergencies or natural disasters based on experiences during the COVID-19 pandemic.

## Figures and Tables

**Table 1:** Mobile Market Organization Demographics.

Geography		
	n	%
Number of States	23	
Northeast	8	35
Midwest	6	26
West	4	17
South	5	22
**Market Operational Status**		
Operating a mobile market for less than two years	9	19
Operating a mobile market for more than two years	39	81
Geographic Location Mobile Markets Serve *		
Urban	42	88
Rural	21	44
Suburban	21	35

* Some organizations served multiple levels of urbanicity so results did not total 100%.

**Table 2:** Demand Change for a Mobile Market from Customers and Community Partners.

	Customers		Community Partners	
	n	%	n	%
More demand for a mobile market program	30	63	28	58
About the same demand for a mobile market program	12	25	14	29
Less demand for a mobile market program	3	6	4	9
Could not make comparison	-	-	2	4
Other	3	6	-	-

**Table 3:** Mobile Market Season Lengths.

2020 Mobile Market Season Length		
	n	%
4–7	23	48
12	10	21
8–11	9	19
<4	6	12
**Length of 2020 MM Compared to 2019**		
Same season length	17	35
Decreased season length	12	25
Increased season length	9	19
Other	1	2
Did not operate a market prior to 2020	9	19

**Table 4:** Changes in Number of Sites Visited in 2020 Compared to 2019.

	n	%
Visited fewer market sites	19	40
Visited more market sites	14	29
Visited approximately the same number of sites	6	12
Did not operate in 2019	9	19

**Table 5:** Prioritization of Sites in 2020 for Markets that Changed Number of Sites.

	n	%
Community demand	23	74
Access	22	71
Food insecurity data	13	42
Income data	4	13

Some organizations selected multiple responses so results did not total 100%.

**Table 6:** Mobile Market Revenue in June, July, August 2020 Compared to 2019.

	n	%
Less	18	37
Increase	8	17
No change	7	15
Uncertain of changes	5	10
Could not make comparison	10	21

**Table 7:** Actions Leading to Lost Revenue During June, July, and August 2020 as Compared to 2019.

	n	%
Decreased customer attendance	17	77
Current funders suspended payments	2	9
Decreased merchandise sales	4	18
Lost sponsors or less income from sponsorships	2	9
Other	3	14

Percentages did not equal 100% since markets may have experienced more than one action leading to revenue loss.

**Table 8:** Actions Taken to Offset Lost Revenue.

Did Your Organization Take Actions to Offset Lost Revenue?		
	n	%
Yes	31	65
Not applicable	17	35
Actions taken to offset lost revenue or increased costs due to COVID-19? *		
Submitted new grants	21	68
Increased funding from existing grants	11	35
Received SBA	11	35
Organized community fundraisers	7	23
Corporate based funding	3	10
Laid staff off	3	10
Took out private loans	1	3
Other	3	10

* Percentages did not equal 100% since markets may have taken one or more actions to offset revenue loss.

**Table 9:** Overall Produce Amount Distributed in 2020 Compared to 2019.

	n	%
Increase in volume of produce distribution	26	65
Approximately the same	8	20
Decrease in volume of produce distribution	6	15

**Table 10:** USDA Farmers to Families Food Box Program Participation.

	n	%
No	32	67
Yes	16	33
Distributed directly	8	50
Distributed outside our Mobile Market	10	63
Delivered to other non-profits to disseminate	5	31
Aggregated food for USDA Farmers to Families Food Box Program	3	19

Percentages did not equal 100% since markets may have participated in the program under multiple modalities.

**Table 11:** Fruit and Vegetable Incentive Program Usage.

Did Your Market See Increase in Use for Any of the Following Types of Fruit and Vegetable Incentive Programs in 2020 Compared to 2019?		
	n	%
Yes	28	58
No	2	4
Did not offer in 2020	10	21
**Incentive programs that increased for markets**		
SNAP/EBT, including P-EBT	20	71
SNAP matching programs	21	75
Produce prescription	9	32
WIC	7	25
Senior Farmers Market Nutrition Program	6	21
Mobile Market Loyalty Program	3	11

Percentages did not equal 100% since markets may have offered more than one incentive program.

**Table 12:** Availability of Pre-Orders or Pre-Payments.

	n	%
No	29	61
Yes, due to COVID-19	16	33
Yes, prior to COVID-19	3	6

## Data Availability

The data presented in this study are available on request from the corresponding author.

## Supplementary Materials

The following supporting information can be downloaded at: https://www.mdpi.com/article/10.3390/ijerph191811390/s1, Supplementary File: Impact of COVID-19 on Mobile Markets in 2020: Snapshot Survey.
