# Supplementary material for: Impacts of the COVID-19 Pandemic on Mobile Produce Market Operations: Adaptations, Barriers, and Future Directions for Increasing Food Access
Source: Int J Environ Res Public Health. 2022 Sep 10;19(18):11390. doi: 10.3390/ijerph191811390 (PMC9517362; doi:10.3390/ijerph191811390)
Supplement: Supplementary file 1 [file ijerph-19-11390-s001.zip › ijerph-1821006-supplementary.pdf]

Impact of COVID-19 on Mobile Markets in 2020: Snapshot Survey

Introduction

- This survey is for organizations that operated a mobile market anytime during 2020.
- The purpose is to capture a quick snapshot mobile market practices and the impact of COVID-19.
- No individual data will be shared by the University at Buffalo; only data that does not identify any individual market organization will be made public.

Demographics

1. What is the name of your organization? [Open Text]
2. Where is your organization located? [Text Entry Zip Code] [State Drop Down]
3. Did your organization operate a mobile market in 2020?
    a. Yes
    b. No [Terminate]
        i. Thank you for interest in this survey. If you were planning to but did not run a mobile market in 2020, please let us know why: ________________. If you have any questions, please reach out to the Veggie Van team at contactus@myveggievan.org. Thank you!

4. How long has your market been in operation? [SLIDER for years]

5. During the 2020 season, how would you describe the geographic location(s) that your mobile market served? Please check all that apply.
    a. Urban
    b. Rural
    c. Suburban
    d. Other (please specify)

Community Served & Operation Duration

6. Since the start of the COVID-19 pandemic, how has the demand for a mobile market program from **customers** changed? [Choose one option]
    a. We are seeing more demand for a mobile market program
    b. We are seeing less demand for a mobile market program
    c. We are seeing about the same demand for a mobile market program
    d. Other (please specify) [Open Text]

7. Since the start of the COVID-19 pandemic, how has the demand for a mobile market program from **community partners** changed? [Choose one option]
    a. We are seeing more demand for a mobile market program
    b. We are seeing less demand for a mobile market program
    c. We are seeing about the same demand for a mobile market program

   d. Other (please specify) [Open Text]

8. How many months out of the year did your mobile market operate in 2020. [SLIDER for months]

9. How did the length of your mobile market season change in 2020 compared to 2019?
   a. Increased season length in 2020 (we operated for more months than in prior years)
   b. Decreased season length in 2020 (we operated for less months than in prior years)
   c. Same season length (we operated for the same number of months compared to prior years)
   d. Not applicable; we did not operate a market prior to 2020.
   e. Other (please specify) [Open Text]

10. How did the number of sites change in 2020 compared to 2019?
    a. Visited less sites in 2020
    b. Visited more sites in 2020
    c. Visited approximately the same number of sites in 2020
    d. Not applicable, did not operate prior to 2020
    e. Other (please specify) [Open Text]

11. If you had to change or reduce the number of sites your operated, how did you prioritize which sites to visit in 2020? Please check all that apply.
    a. Access to sites
    b. Community demand
    c. Food insecurity data
    d. Income data
    e. Not applicable
    f. Other (please specify) [Open Text]

Revenue and Produce Distribution

12. Select the response that best describes your market organization: "Across all market locations, our market sales generated"
    a. LESS revenue during JUNE, JULY, and AUG. 2020 as compared to JUNE, JULY, and AUG 2019
    b. The SAME amount of revenue during JUNE, JULY, and AUG 2020 as compared to JUNE, JULY, and AUG 2019
    c. MORE revenue during JUNE, JULY, and AUG 2020 as compared to JUNE, JULY, and AUG 2019
    d. Not sure what (if any) change in revenue there has been as compared to JUNE, JULY, and AUG 2019
    e. Not applicable; did not run a market in June, July, August of 2019

13. Which actions have led to LOST REVENUE during JUNE, JULY, and AUGUST 2020 as compared to 2019? Please check all that apply.
    a. Not applicable, did not experience decreased revenue during this period.
    b. Decreased customer attendance
    c. Current funders suspended payments (Funders refer to entities that finance all or part of the

market's operations or programming in the form of grants, investments, loans, or other payments. For the purpose of this question, funders do not include sponsorships)
   d. Lost sponsors or less income from sponsorships
   e. Decreased incentive or token sales
   f. Decreased merchandise sales
   g. Other (please specify) [Open Text]

14. What actions, if any, has your organization taken to offset LOST REVENUE or increased costs due to COVID-19? Please check all that apply.
    a. Not applicable, did not experience decreased revenue during this period
    b. Laid off paid staff
    c. Submitted new grant proposals
    d. Increased funding from existing grants (This includes, but is not limited to, requesting additional funds from existing grantmakers and existing grantmakers providing emergency COVID- 19 relief funding)
    e. Took out private bank loans
    f. Received SBA (Small Business Administration) loans or government relief. This includes Paycheck Protection Program (PPP) and Economic Injury Disaster Loans (EIDL)
    g. Organized a community fundraiser for donations
    h. Corporate based funding
    i. None yet
    j. Other (please specify) [Open Text]

15. How does the overall produce amount your organization distributed in 2020 compare to 2019? This would include any produce distributed (e.g., sold, donated). Think about in terms of whatever unit makes sense for your organization (e.g., pounds or boxes of produce):
    a. Increased volume of produce distribution in 2020
    b. Decreased volume of produce distribution in 2020
    c. Approximately the same produce volume distribution in 2020
    d. Not applicable, we did not distribute produce in 2019
    e. Other (please specify) [Open Text]

Incentives

16. Did your market participate in the USDA Farmers to Families Food Box program? Please check all that apply.
    a. No
    b. Yes, we aggregated food for the USDA Farmers to Families Food Box Program
    c. Yes, we distributed USDA Farmers to Families Food Box Program to families at our market sites
    d. Yes, we distributed USDA Farmers to Families Food Box Program to families in another way (not at our mobile market sites)
    e. Yes, we deliver USDA Farmers to Families Food Boxes to other non-profit organizations in the community to disseminate

f. Yes, we had other involvement in the USDA Farmers to Families Food Box Program—please describe [Open Text]

17. Did your market see an increase in use for any of the following types of fruit and vegetable incentive programs in 2020 compared to 2019? Please check all that apply.
    a. SNAP matching program (e.g., Double Up Food Bucks)
    b. WIC Farmers Market Nutrition Program
    c. Senior Farmers Market Nutrition Program
    d. Produce prescription (e.g., Veggie Rx)
    e. Customer loyalty program
    f. SNAP/EBT sales, including P-EBT
    g. Did not offer any of these incentive programs in 2020
    h. Other (please specify) [Open Text]

Operational Changes Made in Response to COVID-19 Restrictions

18. Do you allow pre-orders or pre-payments at your market (by phone, text, or online)?
    a. No, we do not accept orders or sales online, text or phone
    b. Yes, this is something new we added due to COVID
    c. Yes, we offered pre-orders/pre-payments before COVID

19. If Q18=b,c: What types of orders do you accept? Please check all that apply.
    a. Online orders
    b. Text-based orders (please specify) [Open Text]
    c. Phone-based orders
    d. None of the above
    e. Other (please specify) [Open Text]

20. If Q19=a: What is the name of the current online ordering software your organization is using?

21. If Q19=a: How satisfied are you with the online ordering software current in use?
    a. Very satisfied
    b. Somewhat satisfied
    c. Somewhat dissatisfied
    d. Very dissatisfied

22. Did your market experience any of the following barriers while navigating changes due to the pandemic? Please check all that apply.
    a. Secure funding/assistance at the federal level
    b. Secure funding/assistance at the local level
    c. Policies at the federal level
    d. Policies at the local level
    e. Other (please specify) [Open Text]

23. Please describe any additional barriers experienced while trying to operate your market during the pandemic: [Open Text]

24. Where did your organization find information on how to adapt services during the pandemic? [Choose multiple]
    a. Mobile Market Network Listserv
    b. Personal network of other mobile market operators
    c. COVID-19 Open calls hosted by the University at Buffalo's Veggie Van team
    d. Funders
    e. Public Health Departments
    f. Local food access groups
    g. National food access groups/networks (e.g., Farmers Market Association)
    h. Other (please specify) [Open Text]

25. Which innovations, operational "pivots", or adaptations implemented during the COVID-19 pandemic will become long-term or permanent features of your market(s)? [Open Text]

26. Please use this space to provide any additional comments, exciting news, or questions for other mobile markets that you may have. Thank you so much for participating!

Contact Information

27. If you are willing, please share your contact information below so we can possibly follow up with you regarding your responses. Please keep in mind that this survey is anonymous, aside from organization name, and if you share your contact information your answers will no longer be anonymous.
    a. Name [Open Text]
    b. Email Address [Open Text]
    c. Phone Number [Open Text]
